# FLT3 Internal Tandem Duplication and D835 Mutations in Patients with Acute Lymphoblastic Leukemia and its Clinical Significance

**DOI:** 10.4084/MJHID.2014.038

**Published:** 2014-06-01

**Authors:** Ghaleb Elyamany, Mohammed Awad, Omar Alsuhaibani, Kamal Fadalla, Omer Al Sharif, Mohammad Al Shahrani, Fahad Alabbas, Abdulaziz Al-Abulaaly

**Affiliations:** 1 Department of Hematology and Blood Bank, Theodor Bilharz Research Institute.; 2 Dept. of Central Military Laboratory, Prince Sultan Military Medical City, Riyadh, Saudi Arabia.; 3 Dept. of Adult Clinical Hematology and Stem cell Therapy, Prince Sultan Military Medical City, Riyadh, Saudi Arabia; 4 Dept. of Pediatric Hematology/Oncology, Prince Sultan Military Medical City, Riyadh, Saudi Arabia

## Abstract

The fms-like tyrosine kinase 3 (FLT3) gene is a member of the class III receptor tyrosine kinase family. Mutations of FLT3 were first described in 1997 and account for the most frequent molecular mutations in acute myeloid leukemia.

Currently, there is no published data on FLT3 mutations in Saudi acute lymphoblastic leukemia (ALL) patients.

In this retrospective study, we have examined a cohort of 77 ALL patients to determine the prevalence of FLT3 mutations and the possible prognostic relevance of these mutations in ALL patients. Correlations to other biologic factors such as karyotype, molecular mutations, and leukocyte count were also considered.

FLT3 internal tandem duplication (ITD) mutations and point mutation in tyrosine kinase domain (D835) were analyzed in ALL patients, at diagnosis, by polymerase chain reaction (PCR).

Two cases (2.6%, 2/77) were positive for FLT3 mutations; one was found to have FLT3/ITD and the other FLT3/D835.

Our findings suggest that FLT3 mutations are not common in Saudi ALL and do not affect clinical outcome.

## Introduction

**Figure 1:**
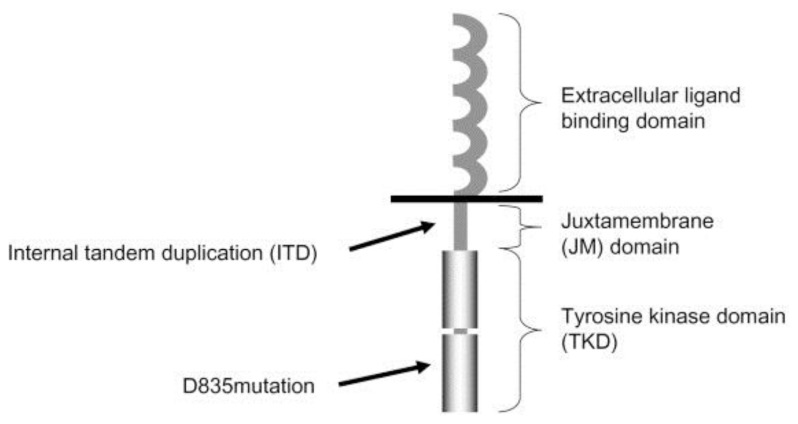
Structure of FLT3 receptor. **Schematic presentation of the FLT3 receptor (**Takahashi S. *Journal of Hematology & Oncology* 2011 **4**:13)

The human fms-like tyrosine kinase 3 (FLT3) gene is located on chromosome 13q12 and encompasses 24 exons. It encodes a membrane-bound glycosylated protein of 993 amino acids with a molecular weight of 158–160 kDa, as well as a non-glycosylated isoform of 130–143 kDa that is not associated with the plasma membrane.^1^ The structure of FLT3 is shown in the figure 1.^2^

Genomic aberrations of FLT3, including internal tandem duplication (ITD) and point mutations, have been demonstrated in approximately 25–35% of adults with acute myeloid leukemia (AML).^3^–^7^ ITD of the FLT3 gene is common in AML and is associated with a bad prognosis and poor response to chemotherapy. Single base mutations at the FLT3 tyrosine kinase domain (TKD), which frequently involves aspartic acid 835 of the kinase domain (D835), leads to a gain of function; however, due to its rarity, its prognostic significance is not well defined.^8^

FLT3 is rarely mutated in leukemic lymphoblasts in adult and pediatric ALL;^3^,^4^,^9^,^10^ however, FLT3 mutations are relatively common among the cytogenetic subgroups of hyperdiploidy and mixed-lineage leukemia (MLL) translocation.^11^

Recent studies have indicated a low overall frequency in childhood ALL (in the 1–8% range) while consistently demonstrating a higher incidence among those with MLL gene rearrangement and high hyperdiploidy.^13^–^17^ In adult ALL, FLT3 mutations are even rarer.^18^

While there have been several studies^19^–^27^ describing activating mutations of the FLT3 gene in AML, there has been little work on these mutations in ALL.

In this study, we analyzed the prevalence of the two types of FLT3 activating mutations in 77 patients with ALL and its prognostic significance. No data currently exist regarding FLT3 mutations in Saudi ALL patients and this study is the first one conducted in Saudi Arabia describing FLT3 mutations in ALL patients.

## Material and Methods

### Study Group

A retrospective review of both adult and pediatric (ages 1 to 15) cases of ALL was performed. Data was obtained from the files of the Department of Hematopathology, Prince Sultan Military Medical City, Saudi Arabia from 2005 to 2013. Leukemia samples were obtained from either bone marrow (BM) or peripheral blood (PB), at diagnosis, from patients with ALL (70 BM samples and 7 PB samples). The peripheral blood samples all had more than 15% blasts at diagnosis. Five samples obtained from normal bone marrow healthy donors were screened for FLT3 mutations as a reference group. Among the 77 patients, with an established diagnosis by cell morphology and flow cytometric immunophenotyping, 48 were pediatric (62.3%), 29 were adult (37.7%), in total, 45 of the patients were male (58.4%) and 32 female (41.6%) Table 1.

Samples were evaluated in addition to cytomorphology, and multiparameter flow cytometry, by cytogenetics, fluorescence in situ hybridization (FISH), and molecular genetics in parallel.

Pediatric patients were treated according to the UKALL 2003 chemotherapy protocol. Initially, eligible pediatric patients were stratified into three risk groups, standard risk (22 patients), intermediate risk (14 patients) and high risk (12 patients) based on age, WBC at presentation, immunophenotype and cytogenetic abnormalities.

Treatment of adult ALL patients was divided into two age groups. Patients at 20 years of age or less were treated according to the Dana-Farber Cancer Institute All Consortium Protocol 00–01^28^ and patients over 20 years of age were treated with Hyper-CVAD chemotherapy.^29^ At first, risk groups at diagnosis were categorized into a standard risk (15 patients) and high risk (14 patients) to determine the intensity of therapy. This study was approved by the Research and Ethics Committee at this institution.

### Morphologic Analysis

For each case in this study, Wright-Giemsa-stained peripheral blood and bone marrow aspirate smears were reviewed. Aspirate clot and biopsy specimens were fixed in formalin, routinely processed and the histologic sections were stained with Hematoxylin and Eosin and reviewed.

### Flow Cytometric Immunophenotypic Methods

All samples were assessed by multicolor flow cytometry using a large panel of antibodies, including CD2, cytoplasmic and surface CD3, CD4, CD5, CD7, CD8, CD10, CD19, CD20, CD22, cytoplasmic CD79a, CD13, CD15, CD33, CD11c, CD14, CD64, CD38, CD34, CD117, cytoplasmic terminal deoxynucleotidyltransferase (TdT), and myeloperoxidase (MPO). Antigens were scored as positive using a cutoff of 20% or more leukemic blasts staining brighter than an isotype-matched negative control.

### Molecular Methods

- Analysis of FLT3-ITD mutation. DNA was extracted using a QIAamp DNA Kit (Qiagen) according to the manufacturer’s recommendations.
  PCR amplification was composed of 200ng of DNA, 50mM KCL, 10mM Tris-HCL, pH8.3, 1.5mM MgCL2, 0.001%(wt/vol) gelatin, 200 μM dNTPs, 0.4μM of each primer (5′-GCAATTTAGGTATGAAAGCCAGC-3′ and 5′-CTTTCAGCATTTTGACGGCAACC-3′), and 1U of gold Taq polymerase, in a volume of 50μl.^23^
  The PCR consisted of an initial incubation step at 95°C for 10 minutes followed by 35 cycles at 94°C for 30 seconds, 57°C for 60 seconds, and 72°C for 90 seconds. The final extension step was at 72°C for 10 minutes on a GeneAmp PCR system 9700(Applied Biosystems). PCR products were analyzed on standard 3% agarose gels. Normal amplification generates a 330bp product; whereas, FLT3 ITD mutations (FLT3/ITD+) show longer PCR products.
- Analysis of the FLT3- D835 mutation. PCR amplification was set up as above using specific primers^4^ for exon 20 (5′-CCGCCAGGAACGTGCTTG-3′ and 5′-GCAGCCTCACATTGCCCC-3′). PCR product was digested with EcoRV (Promega), at 37°C for 2h. The digestion products were separated on a 3% agarose gel, and incomplete digestion indicated the presence of mutant.

### Statistical analysis

The Kaplan-Meier technique was used to analyze the probability of overall survival (OS). OS was calculated from time of diagnosis to death. Continuous variables, such as white blood cell count and hemoglobin, were compared using the Kruskal-Wallis test. Differences between means were considered as significant at P < 0.05.

Complete remission (CR) was defined by less than 5% blast cells in a normocellular marrow and peripheral blood neutrophil count equal to or greater than 1.5 × 10^9^/liter with a platelet count of more than 100 × 10^9^/liter. Normalization of cytogenetic abnormalities was not a prerequisite for CR.

## Results

Clinical characteristics at presentation and cytogenetic analysis of the patients in the studied group are summarized in table 1.

In total, the patient age range was 1 to 81 years with a median of 15 years. Pediatric patients were defined as less than 15 years of age. Of the 77 patients, 48 were pediatric (62.3%), all with de novo ALL (42 cases BALL and 6 cases T-ALL). 29 were adult patients (37.7%) in which there were 27 cases of de novo ALL ( 22 cases B-ALL and 5 cases T-ALL) and 2 cases of CML transforming to ALL. Two cases from the pediatric group were diagnosed as biphenotypic leukemia. In total, 45 of the patients were male (58.4%) and 32 female (41.6%).

**Figure 2:**
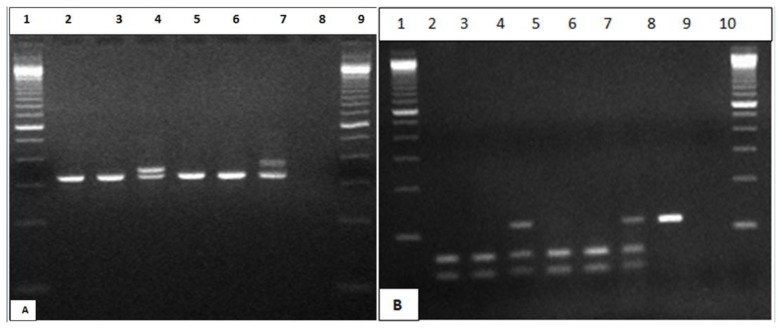
PCR analysis of FLT3-ITD and D835 mutations. A) Agrose gel shows patients positive for ITD lanes 4, patients negative for ITD lanes 2, 3, 5, 6, positive control lane 7, no template control lane 8, Marker lane 1, 9. B) Agrose gel shows digested sample for D835 lanes 2–7, positive patients lane 4 (114bp, 68 bp, 49bp), negative patients lanes 2, 3, 5, 6 (68bp, 49bp), positive control lane 7, Undigested sample lane 8 (114bp), no template control lane 9, Marker lanes 1, 10.

Two of the 77 ALL patients examined showed FLT3 mutations (2/77) with an overall prevalence of 2.6%. Positive FLT3 mutation patients were both pediatric, one male and one female. One was found to have FLT3/ITD and the other was positive for FLT3/D835 (Figure 2). None of the adult ALL patients was positive for FLT3 mutations. None of the T-ALL (T-ALL - 11/77) and CML patients transforming to ALL showed FLT3/ITD or D835 mutations. None of the patients had a combination of FLT3/ITD and D835 mutation in the FLT3 gene.

The FLT3/ITD positive patient (male, 14 years old) showed a WBC of 91 × 10^9^/liter with 70% of the peripheral blasts. The bone marrow (BM) blasts were 95% and showed a good response to chemotherapy resulting in CR. The FLT3/D835 positive patient (female, 4 years old) showed a WBC of 4.6 × 10^9^/liter with 32% of peripheral blasts. The bone marrow (BM) blasts were 95%, and continuous complete remission (CCR) was achieved after chemotherapy.

Cytogenetic and molecular studies revealed that the FLT3/D835 positive patient was hyperdiploidy and reported as 54, XX,+2,+4,+8,+14,+16,+20,21,+21.nuc ish (ABL1, BCR) ×2[100]/(ETV6, RUNX1)x3[95/100]/(5′MLL,3′MLL,5′MLL con 3′MLL)x2[100]/(5′MYC.3′MYC,5′MY con 3′MYC)x3 [95/100]/(TCF3, PBX1)x2[100]. An extra copy of chromosome 2, 4, 8, 12, 14, 16, 20 and 21 was detected in 95% of the studied cells. Cytogenetic analysis could not be performed due to an insufficient number of metaphases.

## Discussion

FLT3 gene mutations, particularly ITD in AML, are well established as the most frequent somatic alterations in AML. A poor prognosis is associated with FTL3 gene mutations in AML and they are found in approximately 5–15% of children and 25–35% of adults with AML.^4^,^7^,^19^,^20^,^23^,^31^ FLT3 mutations are also found in adult and pediatric ALL, but are much rarer than in AML.^3^,^4^,^9^,^10^ FLT3 is overexpressed at the level of RNA and protein in most B lineage and acute myeloid leukemias. It is also overexpressed in a smaller subset of T-ALL and chronic myeloid leukemias (CML) in blast crisis.^32^

This study is the first to report FLT3 mutations on ALL patients in Saudi Arabia. The overall rate of patients with ALL and FLT3 mutations, in this study, was 2.6% which is comparable with previously published reports.^4^,^14^,^33^,^34^,^35^

The incidence of FLT3 mutations in pediatric ALL of this study was 4.2% (2/48). The results are within the range of similar studies conducted in different geographic regions (see table 2).

The incidence of FLT3 mutations in pediatric leukemia is of particular interest due to the several promising FLT3 inhibitors currently under development.^45^

The frequency of both ITD and D835 mutations among adult ALL patients in this study was 0% (0/29); other studies have reported similar results;^19^,^43^,^44^ however, a few studies have reported FLT3 mutations among adult ALL patients at a very low frequency.^18^ As this is a rare occurrence, the limited sample size of this study is the most likely cause of the difference. Similarly, neither FLT3/ITD nor FLT3/D835 mutations were evident in any case of CML transforming to ALL or in T-All (0/11) which is consistent with the study by Anderson et al. 2008 (0% in 15 T-ALL patients).^13^ Some larger studies have reported a low frequency of FLT3/ITD and/or FLT3/D835 mutations ranging from 3.3% to 5.5% among T-ALL patients.^18^,^39^,^46^

In this study, one patient (male, 14 years old) was found to have the FLT3/ITD mutation. He showed leukocytosis and a high blast cell count in PB and BM. This patient exhibited a good response to chemotherapy and achieved complete remission, which is in-line with results of previous studies.^35^,^37^,^39^ This patient was also positive for myeloid antigen expression and was diagnosed as acute mixed-lineage leukemia (biphenotypic leukemia). This observation was reported in other studies^17^,^34^,^35^ which indicates a higher frequency among the biphenotypic group. Cytogenetic analysis could not be performed due to the low number of metaphases.

The other FLT3 mutation patient in this study (female, 4 years old), was found to the FLT3/D835 mutation with no leukocytosis; however, she did show a high blast count in PB and BM. Cytogenetic and molecular studies revealed no association with balanced translocations or MLL gene rearrangement; however, hyperdiploidy (extra copy of chromosome 2, 4, 8, 12, 14, 16, 20 and 21) was detected in 95% of the studied cells. This finding represents a 16.7% incidence of hyperdiploidy in ALL (1/6) cases. Our results reinforced previous observations, Armstrong et al. 2004,^12^ in that the presence of the FLT3 mutation in hyperdiploid ALL did not affect the clinical outcome as the patient responded well to chemotherapy and achieved continuous complete remission (CCR).

In both cases, molecular studies using polymerase chain reaction (PCR) were negative for Philadelphia chromosome [t(9;22)(q34.1;q11.2)].

The only notable difference between this study and previous studies^11^,^12^,^36^ is the lack of association between the high frequency of FLT3 mutation and MLL gene rearrangement. The small sample size (5 ALL patients) is the most likely cause of the difference.

Neither ITD’s or D835 mutations were detected in the healthy donors of this study, and this is consistent with previously reported data.^4^,^47^

## Conclusion

FLT3 mutations exist in a small proportion of Saudi ALL patients. Regarding clinical outcomes, there was no prognostic significance in ALL patients with or without FLT3 mutations. The observation of high frequency in hyperdiploid ALL is in agreement with similar studies from other geographical regions; however, there is a lack of association between MLL gene rearrangement and FLT3 mutations among ALL patient that may be due to the limited sample size. Further studies are needed to confirm and establish these results.

## Figures and Tables

**Table 1:** Clinical characteristics and cytogenetic findings of the patients included in the study.

Parameter	Pediatric ALL (n=48)	Adullt ALL (n=29)
Male:Female	28:20	17:12
Median age (years)	5 (1–14)	31.5 (17–81)
Median WBCs Count (×10^9^/L)	10.500	16.000
Median Platelets Count (×10^9^/L)	37.000	30.500
Median Hb (× gm/dl)	8.2	9.8
Median BM Blasts	90%	70%
Median PB Blasts	40%	32%
5-year survival rate	82%	37%
***FLT3*** **mutations Rate**		
*FLT3*-ITD	1/48 (2.1%)	0/29 (0 %)
*FLT3*-D835	1/48 (2.1%)	0/29 (0 %)
Total	2/48 (4.2%)	0/29 (0 %)
**Cytogenetic Analysis**		
Karyotype (Available)	20 normal 8 abnormal	11 normal 7 abnormal
Available for Fish	n=48	n=28
t (9;22)	4 (8.3%)	6 (21.4%)
t (12;21)	6 (12.6%)	0 (0 %)
MLL	4 (8.3%)	1 (3.6%)
MYC	1 (2.1%)	1 (3.6%)
t (1;19)	1 (2.1%)	1 (3.6%)
+21	6 (12.6%)	0 (0 %)
Del 12p	2 (4.2%)	0 (0 %)
+9	2 (4.2%)	1 (3.6%)
+8	1 (2.1%)	0 (0 %)
−19	2 (4.2%)	1 (3.6%)
Hyperdiploid	3 (6.3%)	3 (10.7%)
tetraploidy	1 (2.1%)	0 (0 %)
Other aberrations	3 (6.3%)	2 (7.1%)

**Table 2:** Frequency of *FLT3* mutations in ALL patients in the current study and in previous studies.

No. of ALL Cases	Mutation number	Mutation (%)	Type of mutation	Comments	Reference
77	2	2.6%	ITD and D835	No prognostic significance value, associated with hyperdiploidy (1/6)	Current Study
90	2	2.2 %	ITD	Adult T-ALL	Grossmann et al, 2013 (37)
	1	1.1 %	TKD		
25	1	4%	ITD	No significant Prognostic value	Ishfaq et al, 2012 (38)
441	9	2%	2 FLT3 ITDs, one deletion mutation, and 6 point mutations	Pediatric group, more common in patients with high hyperdiploidy	Chang et al, 2010 (36)
80	6	7.5%	6 ITD, 0 TKD	Pediatric group, no prognostic difference between FLT3+ and FLT3−	**Karbacak et al, 2010 (35)**
83	2	2.4%	2 D835, 0 ITD,	No Prognostic significance	Wang et al, 2010 (31)
86	2	2.3%	2 AL	Pediatric group, Co-Presence of RAS mutations, high frequency in Hyperdiploid Cases (2/9)	Braoudaki, 2009 (17)
61	3	4.9%	2 ITD, 1 TKD		Zhao et al, 2009 (39)
133	4	3%	3 ITD, 1 D835	Pediatric group, 86 de novo ALL, 37 relapsed ALL	Case M, 2008 (14)
25	0	0%	-	Pediatric group	Al-Tonbary, 2008 (40)
143	8	8%	2 % ITD, 6 % TKD	0% in T-ALL(15), 128 (B-ALL) Pediatric group, High in Hyperdiploid	Andersson et al, 2008 (13)
	
95	1	1%	ITD	Pediatric group	Yamamoto et al, 2006 (16)
27	0	0 %	-	25 ALL, 2 biphenotypic leukemia	Wang et al, 2005 (41)
63	2	3.2%	ITD	2 cases were Biphonotypic leukemia, associated with Poor Prognosis	Xu et al, 2005 (32)
162	14	9%	No ITD, 14 TKD	Pediatric group, high frequency in hyperdiploidy and MLL gene	Taketani et al, 2004 (34)
55	3	5.5%	2 ITD, 1 TKD	Adult T-ALL, 3 cases expressed CD117	Paietta et al, 2004 (18)
36	1	2.8%	TKD	-	Yamamoto et al, 2001 (4)
60	2	3.3%	ITD	2 cases were Biphenotypic, no prognostic value	Xu et al, 1999 (33)
55	0	0%	-	-	Yokota et al, 1997 (19)
50	0	0%	-	-	Nakao et al, 1996 (42)
